# Modelling cost-effectiveness of replacement strategies for ambulance services in the Ministry of Health Malaysia

**DOI:** 10.1186/s12913-024-10557-4

**Published:** 2024-02-06

**Authors:** Nor Zam Azihan Mohd Hassan, Mohd Shahri Bahari, Sivaraj Raman, Farhana Aminuddin, Mohd Shaiful Jefri Mohd Nor Sham Kunusagaran, Nur Amalina Zaimi, Min Fui Wong, Marhaini Mostapha, Ahmad Tajuddin Mohamad Nor, Mohd Ridzwan Shahari

**Affiliations:** 1 Centre of Health Economics Research, Institute for Health Systems Research (IHSR), National Institutes of Health (NIH), Ministry of Health Malaysia, Setia Alam, Shah Alam, Selangor Darul Ehsan Malaysia; 2 Hospital Tengku Ampuan Rahimah, Klang, Selangor Darul Ehsan Malaysia; 3 Medical Development Division, Ministry of Health, Putrajaya, Malaysia

**Keywords:** Ambulance, Cost-effectiveness, Ambulance replacement strategy, Emergency, Markov modelling

## Abstract

**Background:**

Emergency Medical Service (EMS) is a very crucial aspect of the healthcare system in providing urgent management and transportation of patients during emergencies. The sustainability of the services is however greatly impacted by the quality and age of ambulances. While this has led to numerous replacement policy recommendations, the implementations are often limited due to a lack of evidence and financial constraints. This study thus aims to develop a cost-effectiveness model and testing the model by evaluating the cost-effectiveness of 10-year and 15-year compulsory ambulance replacement strategies in public healthcare for the Malaysian Ministry of Health (MOH).

**Methods:**

A Markov model was developed to estimate the cost and outcomes ambulance replacement strategies over a period of 20 years. The model was tested using two alternative strategies of 10-year and 15-year. Model inputs were derived from published literature and local study. Model development and economic analysis were accomplished using Microsoft Excel 2016. The outcomes generated were costs per year, the number of missed trips and the number of lives saved, in addition to the Incremental Cost-Effectiveness Ratio (ICER). One-Way Deterministic Sensitivity Analysis (DSA) and Probabilistic Sensitivity Analysis (PSA) were conducted to identify the key drivers and to assess the robustness of the model.

**Results:**

Findings showed that the most expensive strategy, which is the implementation of 10 years replacement strategy was more cost-effective than 15 years ambulance replacement strategy, with an ICER of MYR 11,276.61 per life saved. While an additional MYR 13.0 million would be incurred by switching from a 15- to 10-year replacement strategy, this would result in 1,157 deaths averted or additional live saved per year. Sensitivity analysis showed that the utilization of ambulances and the mortality rate of cases unattended by ambulances were the key drivers for the cost-effectiveness of the replacement strategies.

**Conclusions:**

The cost-effectiveness model developed suggests that an ambulance replacement strategy of every 10 years should be considered by the MOH in planning sustainable EMS. While this model may have its own limitation and may require some modifications to suit the local context, it can be used as a guide for future economic evaluations of ambulance replacement strategies and further exploration of alternative solutions.

## Introduction

Emergency Medical Service (EMS) is a system consisting of interdependent components, which include pre-hospital care and hospital care. While the main aim is to provide prompt and adequate management of the patient during emergencies, EMS is also pivotal in the management of patient transportation within local and cross-locality healthcare systems [1, 2]. The inability to sustain such services would result in delays in initiating the early crucial treatment and arriving at the nearest healthcare facilities for subsequent management. These delays are known as some of the main reasons for unnecessary preventable death and worsening of the disease prognosis, such as maternal emergency and motor vehicle accidents (MVA) [3].

Compared to the High-Income Countries (HICs), the EMS and pre-hospital services in Lower- and Middle-Income Countries (LMICs), particularly ambulance services are rather in the elementary stage and require further investment to reach their full potential. Most LMICs have given more attention to providing and establishing health facilities to improve healthcare accessibility [4]. This leads to the bulk of the resources being allocated to other sectors instead of the maintenance and renewal of existing ambulances. This forms the main challenge faced by many EMS across the world. Aged and poorly maintained ambulances often lead to the interruption and discontinuation of service. This significantly impacts health service delivery, health outcomes, and the number of lives saved [5]. Furthermore, the limited emergency and ambulance service capacity can lead to the escalation of healthcare costs from more resource-intensive treatment and loss of productivity from premature deaths or disabilities [6].

In Malaysia, the Emergency Response Service 999 (MERS 999) was established in 2007 to coordinate and integrate the ambulance service and the EMS through the Medical Emergency Call Centre (MECC) [7]. Ambulance services in Malaysia were provided by the public and private health providers, wherein the main bulk is provided by the Ministry of Health (MOH). Non-profit agencies such as Saint John Ambulance & Red Crescent Society as well as other government agencies such as the Ministry of Defence (MOD) and Malaysia Civil Defence Department (JPAM) also provide the ambulance service but in much more limited in terms of capacity. Under MOH, the ambulance services were located at the health clinics and hospitals in urban and rural areas to ensure better coverage of pre-hospital care. The function of ambulance services in MOH facilities varies but its main purpose is to transfer patients’ to-and-fro health facilities, either due to emergency cases, trauma or referral of complicated cases. The demand for ambulance services from an upsurge of road traffic accidents and the cases in hospitals and clinics has increased drastically, requiring more reasons to have a sustainable ambulance service [8, 9].

Due to its crucial function in emergencies and prehospital care, the ambulance should be maintained and replaced to ensure its optimal use, patients’ safety and consequently better health outcomes. There are many other potential solutions that may exist to reducing missed-trips and delays, such as increasing fleet size, improving dispatch practices, leveraging private ambulance companies, and others. However, the replacement strategy is one of the important aspects that should be taken into consideration to address insufficient ambulance coverage. Currently, MOH Malaysia has yet to have a proper and documented ambulance replacement strategy. The existing ambulance replacement strategy is mainly adopted as an ad-hoc approach, where any ambulance identified as BER will be replaced irrespective of its age. There is no proper and systematic process of ambulance replacement at the moment. However, the Malaysian Public Works Department (JKR) has suggested the useful life of an ambulance is between 10 and 15 years based on performance and safety reasons, respectively [10]. As the 10-year ambulance replacement strategy may result in a more expensive approach, it is important to explore whether this strategy will be deemed cost-effective to the policymaker. Nevertheless, weighing between ambulance replacements every 10 or 15 years should be done with careful consideration since it will affect the operational budget and require commitment in terms of monetary support. At the same time, the outcome of the ambulance services needs to be improved or at least maintained. Therefore, a cost-effectiveness analysis of ambulance replacement is crucial and should be taken into account in making such an important decision [11].

Commonly used approaches to cost-effectiveness analysis include Random Control Trial (RCT) and decision modelling. While both have their limitations, the decision modelling approach has its upper hand compared to RCT due to its ability to measure more downstream costs and consequences to the implementation of intervention, allowing comparisons of multiple intervention alternatives and comparators, as well as making use of evidence from various sources [12]. Economic modelling is often useful in providing a more pragmatic and transparent approach to economic evaluation in the absence of complete data. Decision modelling attempts to represent reality with just enough details for decision making. The fact that data and information can come from various sources and the results are extrapolated into the future justifies the use of modelling in economic evaluation [13]. A decision tree model and a Markov model are two of the most commonly used decision models in cost-effectiveness analysis. A decision tree model is the simplest model to be used in decision modelling of cost-effectiveness analysis, its main limitation is the unidirectional flow [12]. Hence, only suitable for conditions or disease that follow a particular course such as dead or alive. The Markov model on the other hand is more suitable for certain conditions where a transition from one state to other states are required to understand the course of the disease or condition over a specific time period [12]. It is a simple model to develop and analyse. There are other models such as Discrete Event Simulation and Dynamic Model, but each has its own weaknesses and strengths [13].

Currently, literature on economic analysis of ambulance replacement strategies are very limited. However, the importance of the economic aspect of ambulance replacement strategy cannot be understated. Hence, this study aims to develop a cost-effectiveness model using a Markov model for the ambulance replacement strategy and test the model by evaluating the 10-year and 15-year ambulance replacement strategies. This information would be helpful to provide basic guidance by the MOH for consideration in provision of sustainable ambulance services in MOH.

## Methods

A Markov model was developed to simulate the costs and consequences of a 10-year and 15-year replacement strategy over a period of 20 years, from the perspective of MOH. Most of the costs, outcomes and probabilities were obtained from a local data consisting of 62 health clinics and 14 hospitals in Malaysia, collected from March 2019 to December 2019. The summary of data sources is shown in Table 1. The outcomes generated were costs per year, the number of missed trips and the number of additional lives saved, as well as the Incremental Cost-Effectiveness Ratio (ICER). All costs were presented in Malaysian Ringgit (MYR) and valued in the year 2019, of which MYR 1.00 ~ USD 0.24 [14]. A one-time Gross Domestic Product (GDP) per capita was used as the Willingness-to-pay (WTP) threshold. Malaysia’s GDP per capita was valued at around MYR 40,000 (~ USD 9,600) [15].

**Table 1:** Data and the Source of Data

Data	Type of Data	Source of Data
Cost Data		
Ambulance	Secondary	Engineering Division, MOH
Medical Equipment	Secondary	Engineering Division, MOH Ambulance Records in Hospitals and Clinics in MOH^a^
Personnel	Secondary	Hospitals and Clinics in MOH^a^
Maintenance	Secondary	Engineering Division, MOH Ambulance Records in Hospitals and Clinics in MOH^a^
Fuel	Secondary	Ambulance Records in Hospitals and Clinics in MOH^a^
Ambulance Utilization	Secondary	Ambulance Records in Hospitals and Clinics in MOH^a^
Number of ambulances	Secondary	Medical Development Division, MOH
Mortality rate without ambulance / Mortality rate of patient unattended by ambulance	Secondary	Published literature

^a^Data from 62 health clinics and 14 hospitals in MOH, Malaysia

### Cost-effectiveness model

**Fig. 1:**
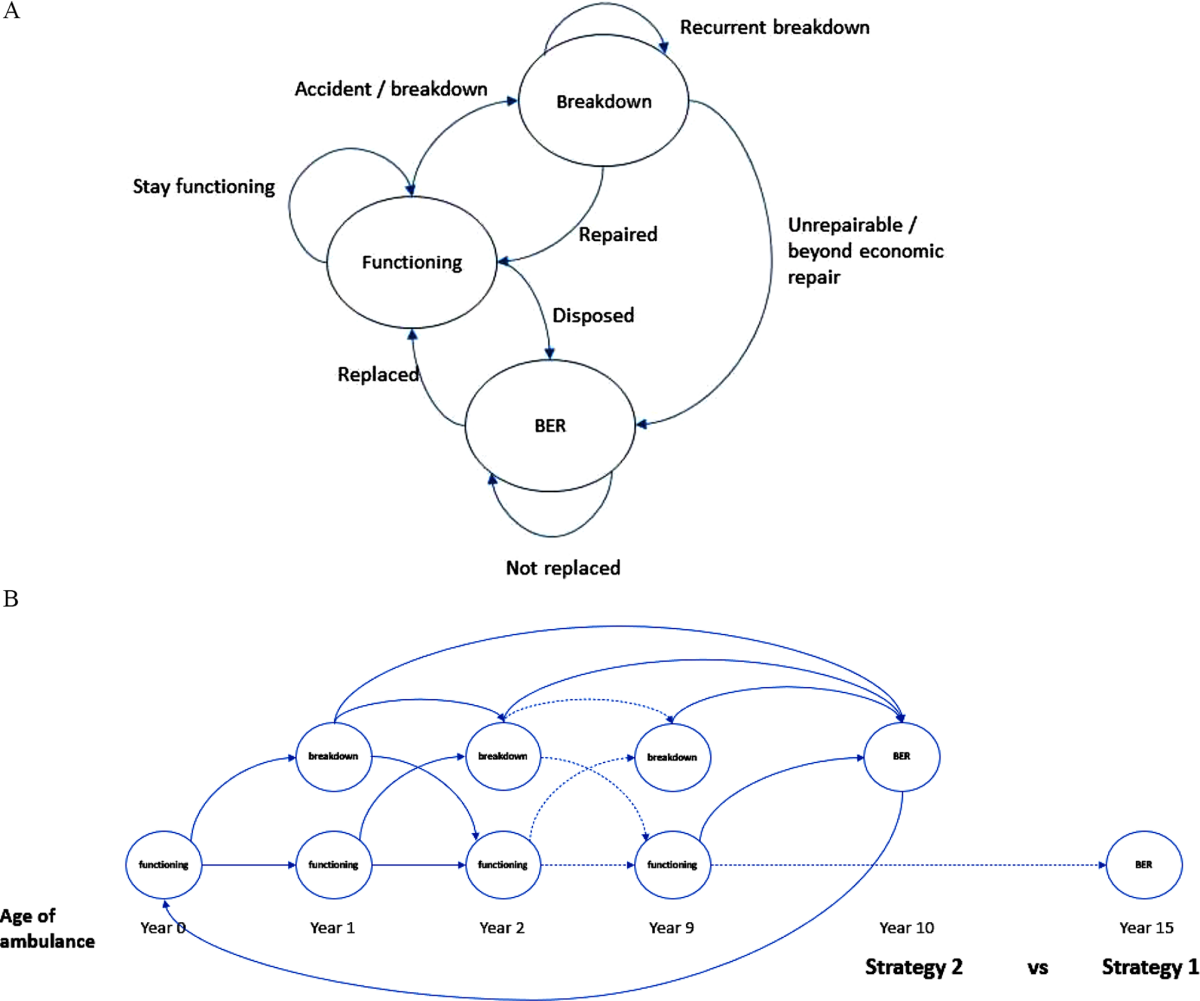
(**A**) Markov Model for Cost Effectiveness of Ambulance Replacement Strategy; (**B**) Transition of Cohort for Each Markov Cycle by Age of Ambulance

A Markov model was developed for the cost-effectiveness analysis using Microsoft Excel 2016 (Fig. 1). Markov model was preferable because of its easily replicable and feasible. The cost-effectiveness model would compare two replacement strategies for ambulance services. Strategy 1 is a replacement of an ambulance every 15 years, while Strategy 2 is a replacement of an ambulance every 10 years. The Markov model was described into three main states, namely, Functioning, Breakdown and Beyond Economic Repair (BER). A total of 1,891 ambulance cohorts was identified based on the number of ambulances provided by the Engineering Division, MOH in the year 2019 [16]. The ambulance cohort was placed in the model according to their respective age (Fig. 1B). Each of the states would move to other states based on the transitional probabilities of its respective age. Each cycle was set at a 1-year duration, and the model was allowed to run for 20 cycles.

The following shows the mathematical equation of each state. The functional state, *F* is expressed as:

$$ {F}_{t}=\left({F}_{t-1}\times {TP}_{FF}\right)+\left({B}_{t-1}\times {TP}_{BF}\right)+{E}_{t-1}$$

Where

*F*_*t*_ is the number of ambulances in the functional state at time t;

*F*_*t−1*_ is the number of ambulances in the functional state at time t-1;

*B*_*t−1*_ is the number of ambulances in the breakdown state at time t-1;

*E*_*t−1*_ is the number of ambulances in the BER state at time t-1;

*TP*_*FF*_ is the transitional probability to stay at functional state (functional state to functional state) for the respective ambulance age;

*TP*_*BF*_ is the transitional probability from breakdown state to functional state for the respective ambulance age;

The breakdown state, B is expressed in the following equation:

$$ {B}_{t}=\left({B}_{t-1}\times {TP}_{BB}\right)+\left({F}_{t-1}\times {TP}_{FB}\right)$$

Where,

*B*_*t*_ is the number of ambulances in the breakdown state at time t;

*TP*_*BB*_ is the transitional probability to stay at breakdown state (breakdown state to breakdown state) for the respective ambulance age;

*TP*_*FB*_ is the transitional probability from functional state to breakdown state for the respective ambulance age;

Finally, the BER state is expressed using the equation below:

$$ {E}_{t}=\left({F}_{t-1}\times {TP}_{FE}\right)+\left({B}_{t-1}+{TP}_{BE}\right)$$

Where,

*E*_*t*_ is the number of ambulances in the BER state at time t;

*TP*_*FE*_ is the transitional probability from functional state to BER state for the respective ambulance age;

*TP*_*BE*_ is the transitional probability from breakdown state to BER state for the respective ambulance age.

This cost-effectiveness model was carried out based on a few assumptions. First, all BER ambulances will be replaced in the following year. Second, those ambulances in the BER state were assumed to be immobile, hence, they will not be utilized in the current year. All ambulances were presumed to have similar fuel consumption and personnel requirements. It was also assumed that each ambulance trip would only carry one patient while each breakdown will half the operational capacity of a fully functional ambulance since the ambulance will be at the workshop for some period of time to be fully repaired.

### Ambulance data

It was assumed that the number of ambulances retrieved is the optimum number of ambulances for the MOH and there will be no changes in the subsequent years. The ambulance data were grouped by age of five years and less, six to ten years and more than ten years. The transition probabilities were estimated based on the available secondary data retrieved from the Engineering Division, MOH Malaysia. Ambulance utilization data was retrieved from the transport logbook of 62 selected health clinics and 14 hospitals in MOH, Malaysia. To estimate the missed trips by the ambulances, the optimum number of ambulance utilization was set according to the number of utilization of ambulances of age five years and less, making the assumption that ambulances in this age group have met all the ambulance demand and all ambulances should achieve that target number of utilizations. To run the model, it was assumed that there was no alternative ambulance service and patients would be transported by other means such as family members, passer-by and others, rather than ambulance services. Hence, the missed trips or ambulance non-conveyance were estimated from the optimum ambulance utilization per year subtracted from the total number of ambulance utilization.

The mortality rate without an ambulance was estimated based on the risk of mortality if the patient is not attended or not conveyed by ambulance [3]. According to the literature, the mortality due to non-conveyance is around 2.5–6.1% [3]. This study will use the middle point, of which 4.0% for the analysis. This takes into the assumption that patients will take other means of transportation other than ambulances such as by family members, friends, passer-by and others. Ambulance missed trips multiplied by this mortality rate would result in the estimated total number of mortalities in case of non-conveyance. The number of such deaths averted was set as additional lives saved and used as the effectiveness measure in this cost-effectiveness study. Details of ambulance data used in the analysis are shown in Table 2.

**Table 2:** Ambulance data for the Markov model

Variables	Values	Range	Alpha	Beta	Distribution
**Transitional Probabilities ≤ 5 years**					
Functioning to functioning	0.980				
Functioning to breakdown	0.020	0.016–0.024	24.480	1199.520	Beta
Functioning to BER^a^	0.000				
Breakdown to breakdown	0.250				
Breakdown to functioning	0.375				
Breakdown to BER^a^	0.375	0.300–0.450	15.250	25.417	Beta
BER^a^ to Functioning (replacement)	1.000				
**Transitional Probabilities 6–10 years**					
Functioning to functioning	0.893				
Functioning to breakdown	0.087	0.070–0.104	22.738	238.618	Beta
Functioning to BER^a^	0.020				
Breakdown to breakdown	0.161				
Breakdown to functioning	0.129				
Breakdown to BER^a^	0.710	0.568–0.852	6.540	2.671	Beta
BER ^a^ to Functioning (replacement)	1.000				
**Transitional Probabilities > 10 years**					
Functioning to functioning	0.525				
Functioning to breakdown	0.378	0.302–0.454	15.172	24.966	Beta
Functioning to BER^a^	0.097				
Breakdown to breakdown	0.016				
Breakdown to functioning	0.141				
Breakdown to BER^a^	0.843	0.674–1.000	3.082	0.574	Beta
BER^a^ to Functioning (replacement)	1.000				
**Effectiveness Data**					
Ambulance utilization (number of trips) per year					
≤ 5 years	555	444–666			Gamma
6–10 years	385	308–462			Gamma
>10 years	294	235–353			Gamma
Optimum utilization per year^b^	555				
Mortality rate without ambulance^c^	0.04	0.032–0.048			Gamma
**Other ambulance data**					
Number of Minor Maintenance per year					
≤ 5 years	3				
6–10 years	3				
> 10 years	5				
Number of Major Maintenance per year	1				

^a^BER stands for Beyond Economic Repair

^b^Optimum utilization per year is based on the number of utilization of ambulances of age five years and less

^c^Mortality rate of patient unattended by ambulance [3]

### Costs estimate

The cost data were estimated from the perspective of the healthcare provider. This study incorporates the direct cost of ambulance services using secondary data from multiple sources (Table 1). The cost of ambulance services consisted of ambulance price, medical equipment price, personnel cost, fuel cost, maintenance cost, repair cost and scrapped (or resale) value. Ambulance and medical equipment prices were based on the replacement cost of the year 2019. The scrapped or resale value was estimated using 10% of the ambulance purchasing price [17, 18]. The cost parameters are shown in Table 3. Below is the mathematical equation to derive the total annual cost of an ambulance. The total operating cost per year, *TC*_*o*_ is estimated using the following equation:

$$ {TC}_{o}=\left[U\times \left({C}_{p}+{C}_{f}\right)\times F\right]+\left[U\times \left({C}_{p}+{C}_{f}\right)\times 0.5B\right]$$

Where,

*U* is ambulance utilization (number of trips per year);

*C*_*p*_ is personnel cost per trip;

*C*_*f*_ is the fuel cost per trip;

*F* is the number of ambulance functioning;

*0.5B* is the number of ambulance breakdown with half cycle correction applied;

The total maintenance cost per year, *TC*_*m*_ is shown in equation below:

$$ {TC}_{m}=\left({C}_{mi}\times {M}_{mi}\right)+\left({C}_{ma}\times {M}_{ma}\right)$$

Where,

*C*_*mi*_ is the cost of minor maintenance;

*M*_*mi*_ is the number of minor maintenance per year;

*C*_*ma*_ is the cost of major maintenance;

*M*_*ma*_ is the number of major maintenance per year;

The number of minor maintenances, *M*_*mi*_ required is

$$ {M}_{mi}=\left(F\times {N}_{mi}\right)+\left(0.5B\times {N}_{mi}\right)$$

Where

*F* is the number of ambulance functioning;

*0.5B* is the number of ambulance breakdown with half cycle correction applied;

*N*_*mi*_ is the number of minor maintenances required per year;

The number of major maintenances, *M*_*ma*_ required is

$$ {M}_{ma}=F+B$$

Where

*F* is the number of ambulance functioning;

*B* is the number of ambulance breakdown;

*N*_*mi*_ is the number of major maintenances required per year;

The total repair cost per year, *TC*_*w*_ is shown in the following equation:

$$ {TC}_{w}={C}_{w}\times B$$

Where,

*C*_*w*_ is the cost of ambulance repair;

*B* is the number of ambulance breakdowns;

The total replacement cost per year, *TC*_*r*_ was estimated using the following equation:

$$ {TC}_{r}=R\times \left({C}_{a}+{C}_{e}\right)$$

*R* is the number of ambulances replaced;

*C*_*a*_ is the replacement cost of the ambulance;

*C*_*e*_ is the replacement cost of medical equipment;

The total resale value, *TS* was calculated using the equation below:

$$ TS=S\times E$$

Where,

*S* is resale or scrapped value;

*E* is the number of BER ambulances.

Finally, the total cost of an ambulance per year, *TC* was estimated as below:

$$ TC={TC}_{o}+{TC}_{m}+{TC}_{w}+{TC}_{r}-TS$$

Where,

*TC*_*o*_ is the total operation cost per year;

*TC*_*m*_ is the total maintenance cost per year;

*TC*_*w*_ is the total repair cost per year;

*TC*_*r*_ is the total replacement cost per year;

*TS* is the total resale value per year;

**Table 3:** The cost parameters for ambulance services

Cost components		Costs (MYR)	Range	Distribution
Ambulance price		300,175.00	240,140.00–360,210.00	Gamma
Medical equipment price		188,825.00	151,060.00–226,590.00	Gamma
Personnel	per ambulance per trip	123.99	99.19–148.79	Gamma
Fuel	per ambulance per trip	18.84	15.07–22.61	Gamma
Maintenance	minor	481.69	385.35–578.03	Gamma
	major	1,408.48	1,126.78–1,690.18	Gamma
Repair	per breakdown per year	5,550.98	4,440.78–6,661.18	Gamma
Resale value		30,017.50	24,014.00–36,021.00	Gamma

### Sensitivity analysis

#### Deterministic sensitivity analysis

A one-way sensitivity analysis was performed to determine the uncertainty in input parameters. Each parameter was varied by an increment and decrement by 20% of the values provided in Tables 2, 3 and 4. Any changes in ICER value were recorded and presented as a Tornado diagram. The Tornado diagram is useful in demonstrating the key drivers affecting the ICER based on the level of changes in economic conclusions.

### Probabilistic sensitivity analysis

Probability Sensitivity Analysis applied a Bayesian method to measure the effect of parameters’ uncertainty. All the parameters were allowed to vary according to their appropriate distribution model. The effects were demonstrated by running 10,000 simulations. Subsequently, WTP thresholds were varied up to MYR 120,000 (three times GDP per capita) to test the cost-effectiveness at different WTP thresholds. The graphical presentation of the results was presented as a cost-effectiveness plane scatter diagram and Cost-Effectiveness Acceptability Curve (CEAC).

### Ethics

This study was conducted in compliance with the tenets of the Declaration of Helsinki. This study was registered under National Medical Research Register (NMRR), MOH Malaysia (NMRR-18-2944-44909) and received ethical clearance from Medical Research and Ethics Committee (MREC), MOH Malaysia. All data used in this study received written approval from the data owner (Engineering Department, Medical Development Division, Family Health Development Division and respective State Health Departments, Ministry of Health Malaysia) and the ethics committee.

## Results

Table 4 shows the results of the cost-effectiveness analysis of ambulance replacement Strategy 2 (ambulance replacement every 10 years compared) to Strategy 1 (ambulance replacement every 15 years). Findings showed that implementation of Strategy 1 would incur a lower cost of MYR 153.9 million compared to Strategy 2 of MYR 166.9 million per year. Despite that, adopting Strategy 1 would result in 235,140 ambulance trips missed, causing an estimated 8,230 deaths yearly due to patients unattended by ambulances. The number of missed trips and deaths due to patients unattended by ambulances however could be reduced to 202,080 and 7,073, respectively by adopting Strategy 2. Therefore, even though an additional MYR 13.0 million would be incurred by switching from Strategy 1 to Strategy 2, there would also be an additional 1,157 deaths averted or lives saved per year. At a WTP threshold of MYR 40,000, strategy 2 with an ICER of MYR 11,276.61 per life saved was deemed to be cost-effective.

**Table 4:** The Cost-Effectiveness of Ambulance Replacement Strategy 2 vs. Strategy 1

	Strategy 1	Strategy 2	Incremental
**Costs**	153,888,999.21	166,938,858.63	13,049,859.42
Total operation cost per year, MYR	85,417,235.51	89,928,351.78	4,511,116.27
Total maintenance cost per year, MYR	3,793,496.91	3,762,545.81	-30,951.10
Total repair cost per year, MYR	531,427.53	260,722.05	-270,705.48
Total replacement cost per year, MYR	68,576,975.73	78,067,288.36	9,490,312.63
Total resale value per year, MYR	4,430,136.46	5,080,049.36	649,912.90
** Total cost per year, MYR** ^**a**^	**153,888,999.21**	**166,938,858.63**	**13,049,859.42**
**Effectiveness**			
Missed trips per year	235,140	202,080	-33,060
Death per year^b^	8,230	7,073	-1,157
** Lives saved per year** ^**c**^	-	-	**1,157**
**ICER**	-	11,276.61	-

^a^Total cost per year is calculated by summing up the costs of operation, maintenance, repair and replacement and subtracting the resale value

^b^Based on the mortality rate of patient unattended or non-conveyed by ambulance [3]

^c^life saved is defined as the number of deaths averted, which is the opposite of incremental death

### Sensitivity analysis

**Fig. 2:**
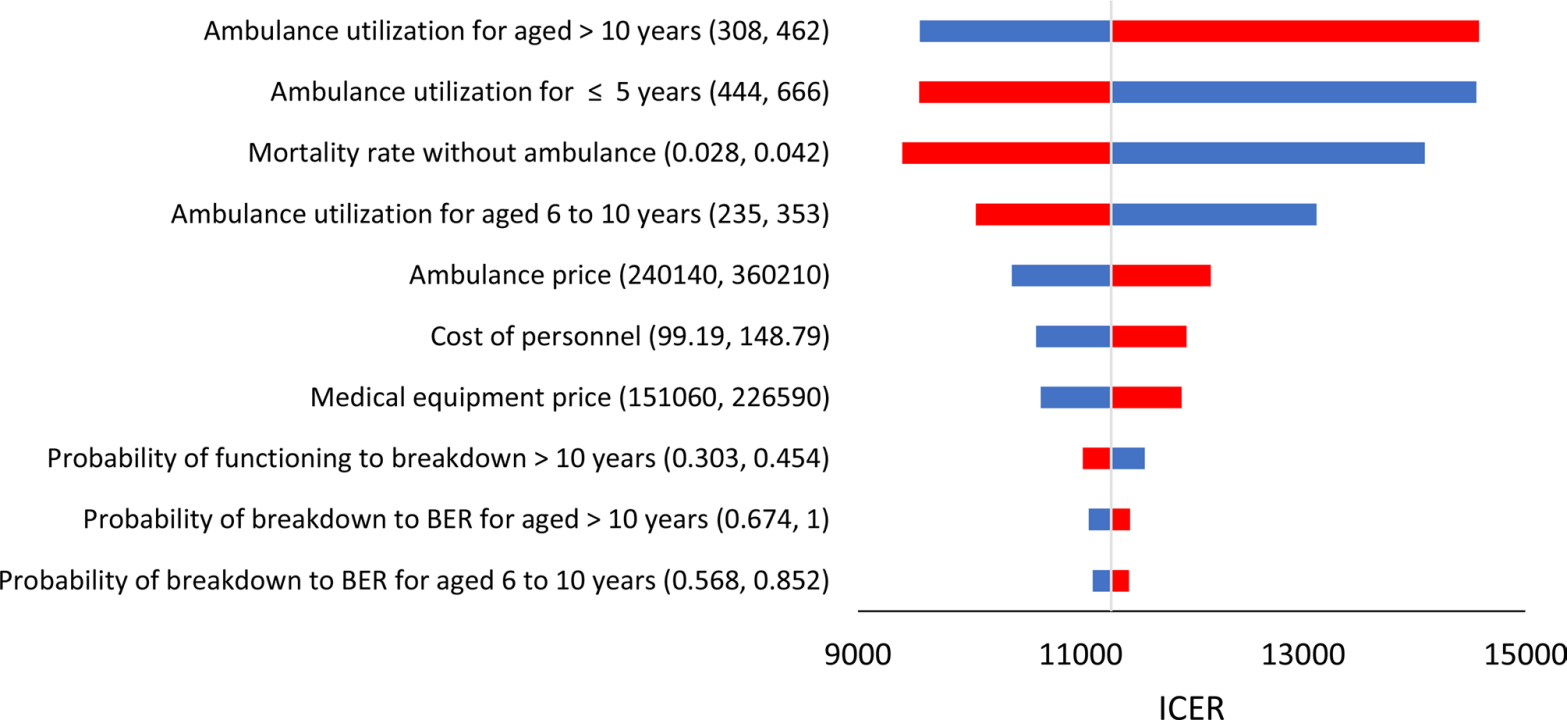
One-Way Deterministic Sensitivity Analysis of Cost-Effectiveness Ambulance Replacement Strategy 2 vs. Strategy 1 visualized in a Tornado Diagram

One-Way DSA for ambulance service replacement Strategy 2 against Strategy 1 is shown in the tornado diagram (Fig. 2**)**. Even at various ranges, the top 10 parameters that affected the ICER values did not change the decision on the cost-effectiveness of strategy 2. The mortality rate of emergency cases not attended by ambulance and ambulance utilization were the main key drivers for the ICER. Repair costs, maintenance costs and fuel costs only have minimal effect on ICER.

**Fig. 3:**
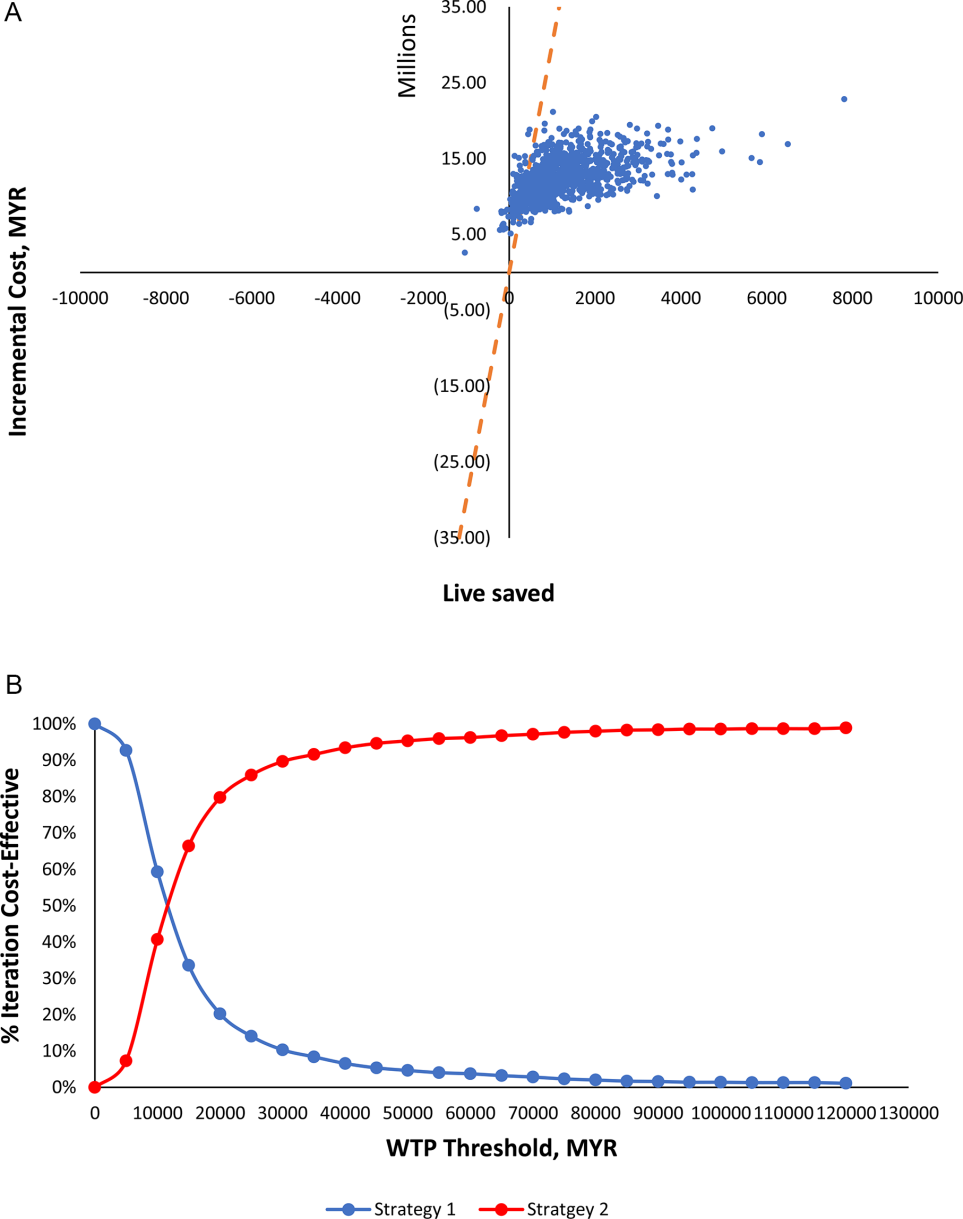
Probabilistic Sensitivity Analysis of Ambulance Replacement Strategy 2 vs. Strategy 1; (**A**) Cost-effectiveness scatter diagram; (**B**) Cost-Effectiveness Acceptability Curve

Figure 3 shows the results of PSA for the cost-effectiveness of ambulance replacement Strategy 2 versus Strategy 1. The results showed that the cost of Strategy 2 was always more expensive than Strategy 1 as all iterations occupied the upper half of the plane (Fig. 3A). However, 98.7% of the iterations were placed in the northeast quadrant (quadrant I), while the remaining 1.3% were in the northwest quadrant (quadrant IV). This means that 98.7% of the time Strategy 2 was more effective and can save more lives than Strategy 1. The CEAC in Fig. 3B evidenced that at a WTP of MYR 12,000 and above, strategy 2 was more cost-effective than Strategy 1.

## Discussions

The cost-effectiveness analysis using the developed Markov chain simulation can be very useful for policymakers in deciding the best strategy to adopt in planning for ambulance replacement. Markov model allows for the simulation of events to mimic the real-world scenario using the local data. This is especially critical for issues involving sequential and stochastic decisions over time. Markov chain simulation was preferable due to its feasibility and replicability [19]. Other approaches such as using decision trees might be limited by the robustness of findings as it over-simplifies the complexity of the consequences of the decisions [20]. While the Discrete Event Simulation (DES) model is more flexible and offers retention of patient history and risk profile updates for each event, it is much more complicated and not readily replicable compared to the Markov model [19, 21]. Decision models such as the decision tree are too simplistic, while the dynamic model requires advanced knowledge in statistics or programming and usually more preferable in infectious disease modelling [13]. Though important, choosing the model needs to weigh their pros and cons, at the same time very much dependent on the data availability.

The findings of this study suggest that the more expensive strategy, wherein ambulance replacement every 10 years are more cost-effective than ambulance replacement every 15 years. The findings were in line with the proposition made by the JKR that the useful life taking into account the performance and economic value of the ambulance is 10 years [10]. The study revealed that the additional costs incurred by switching from the ambulance replacement strategy of 15 years to 10 years were mostly due to the additional operational and replacement costs. Since ambulances will be replaced more often and ambulances will be utilized more frequently, it is no surprise that both costs would escalate. An ambulance utilization analysis in Malaysia further evidenced that newer ambulances had higher utilization in terms of duration and the number of trips compared to those above 10 years [22]. Despite 10 years of ambulance replacement approach being the more expensive strategy of the two, the cost-effectiveness analysis reflected the opposite finding. The outcome of the ambulance in term of missed trips and mortality outweigh the additional cost incurred.

As the vehicle ages, various issues on vehicle performance and safety will arise, particularly in terms of vehicle quality, engine performance, motor power, fuel consumption, structural integrity that involve higher operational and maintenance cost. Even though there were cost reductions observed in Strategy 2, the expected lower maintenance and repair costs were still not sufficient to compensate for the higher gross expenditures for the purchase of more ambulances. However, it is worth noting that maintenance also affects the safety of the ambulance and their number of trips which can result in a higher accident and breakdown rate [23]. This may jeopardize the safety of patients and paramedics in the long run.

Additionally, since aged ambulances would be utilized less due to frequent breakdowns and performance issues, they may no longer be cost-efficient to be maintained. This inadvertently results in patients not being attended by ambulance or not getting proper pre-hospital care, hence leading to a higher chance of mortality [3]. Therefore, reducing the period for replacement of ambulances from 15 years to 10 years could avoid this situation and indirectly improve the pre-hospital care services and be able to save more lives.

Although the utilization of ambulance services was the main driver for the ICER, it did not have much effect on the cost-effectiveness of the replacement strategy. Improvement in the utilization of ambulances in particular those ambulances aged more than 10 years will reversely affect the cost-effectiveness, leading to a more favourable outcome for the replacement strategy every 15 years. The outcomes of this study and subsequent decision-making for replacement may be impacted by improved ambulance quality and structural integrity, which are essential in maintaining ambulance operation and durability [24]. Similarly, improvement in pre-hospital care such as the availability of Automated External Defibrillator (AED) and bystander skills for CPR may improve the outcomes for trauma and cardiac arrest patients [25, 26]. However, this would require significant improvement in pre-hospital care and minimal improvement will not budge the ICER value. Not to mention, the quality of management at the pre-hospital and hospital level also can affect the outcome of these cases conveyed or not-conveyed by the ambulance. Besides, the study also did not take into account the ambulance services provided by the private and non-government health care providers due to the lack of data availability. Inclusion of ambulance services from these other providers would probably affect the outcome of the study. However, the sensitivity analysis conducted showed that there are not much changes in terms of the cost-effectiveness (ICER) despite variation of the mortality rate due to the non-conveyance.

The current study is using one GDP per capita as the WTP threshold and the Value of Statistical Life (VSL) as the cut-off point for the Cost-Effectiveness threshold [27]. By varying the WTP value, it was shown that as long as the WTP threshold is set to at least MYR 12,000, the ambulance replacement strategy every 10 years is deemed as the cost-effective approach. This value is less than 0.5 GDP per capita and is less than the Malaysia CE threshold valuation made in 2014, which is MYR 29,080 [27, 28]. Hence, the WTP value used will not have much effect on the results of this current study, unless society’s valuation of life is significantly reduced, which is very unlikely. Thus, the policymakers need to decide on the WTP value or the CEA threshold prior to decision making. While the value of statistical life is not available in Malaysia and no documented WTP threshold to be used for life saved, this value is assumed to be at least similar or probably higher than the WTP threshold currently used of 1 times GPD per capita.

This study has several limitations, with no measurement on the existing ambulance replacement strategy in MOH Malaysia due to a lack of documentation and data on the current practice. The existing ambulance replacement strategy is mainly adopted as an ad-hoc approach, where any ambulance identified as BER will be replaced irrespective of its age. Some ambulances are still in use despite being more than 15 years old and some ambulances were still not replaced despite being in the BER state [22]. This study also did not include the variability of ambulance services between urban and rural as well as hospitals and clinics. Decision makers may want to have a different approach for different geographical locations since the utilization frequency of ambulances in rural facilities differs compared to urban [22].

This is the first study documenting the cost-effectiveness of an ambulance replacement strategy in the region. The studies that are now available typically compare the different types of emergency modalities such as drones, motorcycles or helicopters versus the conventional available ambulances [29, 30]. This study seeks to address insufficient ambulance coverage, which currently leads to substantial missed trips (and likely delayed pickups), which leads to substantial ongoing mortality. While there are other potential solutions that may exist to reducing missed-trips and delays, such as increasing fleet size, improving dispatch practices, leveraging private ambulance companies, and others. This study analyzes one solution, namely the ambulance replacement interval policy. The study established a novel modeling technique that could be emulated in other jurisdictions that are interested in analyzing how their replacement strategies could be optimized, with an end towards decreased missed-trips and thereby lowering mortality.

This study also highlights the importance of monetary commitment from the government in sustaining ambulance services. Besides, proper planning and strategy are required to ensure that the ambulance services are uninterrupted and sustainable in the long run. The interruption of ambulance service can cause a significant impact on patient outcomes. Future studies should explore the various factors that can affect ambulance service replacement strategies and incorporate those factors into the model.

## Conclusions

In conclusion, this study found that the cost-effectiveness model developed may provide a basic platform for other jurisdictions that are interested in economic assessment of ambulance replacement strategy. While the finding showed that the more expensive strategy, of which replacing the ambulance every 10 years is more cost-effective than the 15 years replacement strategy, it should be taken with caution as there is a limitation with the model used in the study as well as few assumptions that were made during the analysis and model developments. Nevertheless, this opens up new possibilities in assessing ambulance replacement strategies. The model can be improved and expanded in the future by rectifying the limitation whilst exploring the various factors that contribute to ambulance services and incorporating them into the model.

## Data Availability

The data used and generated in this study is not publicly available due to confidentiality issues of the MOH data. The data may be requested from the corresponding author on reasonable request and with permission from the Director General of Health, Malaysia. Data provided/used was anonymized before use.

## Abbreviations

AED: Automated External Defibrillator
BER: Beyond Economic Repair
CEAC: Cost-Effectiveness Acceptability Curve
CPR: Cardiopulmonary Resuscitation
DES: Discrete Event Simulation
DSA: Deterministic Sensitivity Analysis
GDP: Gross Domestic Product
HICs: High-Income Countries
ICER: Incremental Cost-Effectiveness Ratio
JKR: Malaysian Public Works Department
LMICs: Lower- and Middle-Income Countries
MOH: Ministry of Health
MVA: Motor vehicle accident
MYR: Malaysian Ringgit
OHCA: Out-of-Hospital Cardiac Arrest
PSA: Probabilistic Sensitivity Analysis
VSL: Value of Statistical Life
WTP: Willingness-To-Pay
